# Supplementary material for: Prediction model of land surface settlement deformation based on improved LSTM method: CEEMDAN-ICA-AM-LSTM (CIAL) prediction model
Source: PLoS One. 2024 Mar 7;19(3):e0298524. doi: 10.1371/journal.pone.0298524 (PMC10919871; doi:10.1371/journal.pone.0298524)
Supplement: Attachment — Submitted filename: Responses to Reviewers.doc [file pone.0298524.s008.doc]

**Responses to Reviewers’ Comments for the paper entitled**

**Prediction model of land surface settlement deformation based on improved LSTM method:** **CEEMDAN-ICA-AM-LSTM (CIAL) prediction model**

PLoS One ·

by Shengchao Zhu, Yongjun Qin, Xin Meng, Liangfu Xie, Yongkang Zhang, Yangchun Yuan

The authors wish to thank the editor and reviewers for their very thorough, insightful, and constructive reviews. All comments have been addressed in the revised version of the manuscript. We have adjusted our manuscript according to the journal’s requirements. Information regarding funding support has been supplemented in the cover letter. Data and code related to the manuscript have been submitted in separate files. Below are our responses to the questions raised by the academic editor and reviewers.

Academic editor:

1. Improve the structure of the manuscript.

*Thank the editor for the valuable comments on the article. We have refined the structure of the article in the style of PLoS One.*

2. Recheck all equations.

*Thank you for your reminding. We've checked all the equations and made sure they're correct.*

3. Explain evaluating indicators and the reason to use them.

*Thank the editor for the valuable comments. We have already discussed the evaluating indicator in Section 3.2 and explained their meaning with equations.*

4. Provide more clarity on model training. How dataset was organized and what approach was followed. Provide the flowchart.

*Thank the editor for the valuable comments.* *The training process and specific methods of the model have been provided in the paper, as shown in Fig 11. (Line 353)*

5. While conclusion is provided, also provide managerial implications, recommendations, and limitations.

*Your suggestion really means a lot to us.* *The managerial implications, recommendations, and limitations have been provided after the conclusion. (Line 442~446)*

Reviewer #1:

1. Error at line 55 "Moghaddasi et al.Error! Reference source not found.[10] utilized", line 137, line 304, line 309, line 314, line 386, and line 394.

*Thanks for your careful checks.* *We have reinserted the reference* [10] *(Line 55) to ensure it is discovered. We have fixed the errors in line 137, line 309, line 314, line 386 and line 394. We apologize for not finding an error in line 304.*

2. Avoid to use number at you conclusion such as 1,2,3. Replace it with for example "First, second, third" and so on.

*Thanks for your suggestions. We have replaced the number with "First, second, third" and so on. (Line 424,431,436)*

Reviewer #2:

1. In Section 4.1 Performance comparison:

There is no baseline or featureless model.

For regression tasks, a baseline model always predicts the mean of the training set ignoring any input features.

*Thank the reviewer for the valuable comments.* *The data obtained in the experiment already possesses temporal characteristics, and since our model does not contain other features, we believe that there is no need to compare it with a featureless model.*

2. In Section 3.2 Evaluating Indicator Section:

RMSE, MAPE and the other indicators mentioned there are known widely in this field.

There is no need to explain in detail and write formulas.

*Thank you again for your positive comments and valuable suggestions to improve the quality of our manuscript.* *The description of the evaluation indicators in Section 3.2 is provided to account for the specific indicators used in this paper to assess the model, hence the need for explanation.*
